# Understanding the impacts of coastal deoxygenation in nitrogen dynamics: an observational analysis

**DOI:** 10.1038/s41598-024-62186-w

**Published:** 2024-05-23

**Authors:** Laura Farias, Lucas de la Maza

**Affiliations:** 1 Departamento de Oceanografía, Facultad de Ciencias Naturales y Oceanográficas, Universidad de Concepción, Concepción, Chile; 2 Instituto Milenio de Socio-Ecología Costera (SECOS), Santiago, Chile; 3 Center for Climate and Resilience Research (CR), Santiago, Chile

**Keywords:** Coastal upwelling, Fixed nitrogen loss, Air-sea N_2_O flux, Temporal and interannual variability, ENSO, Marine chemistry, Physical oceanography

## Abstract

Biological production and outgassing of greenhouse gasses (GHG) in Eastern Boundary Upwelling Systems (EBUS) are vital for fishing productivity and climate regulation. This study examines temporal variability of biogeochemical and oceanographic variables, focusing on dissolved oxygen (DO), nitrate, nitrogen deficit (N deficit), nitrous oxide (N_2_O) and air-sea N_2_O flux. This analysis is based on monthly observations from 2000 to 2023 in a region of intense seasonal coastal upwelling off central Chile (36°S). Strong correlations are estimated among N_2_O concentrations and N deficit in the 30–80 m layer, and N_2_O air-sea fluxes with the proportion of hypoxic water (4 < DO < 89 µmol L^−1^) in the water column, suggesting that N_2_O accumulation and its exchange are mainly associated with partial denitrification. Furthermore, we observe interannual variability in concentrations and inventories in the water column of DO, nitrate, N deficit, as well as air-sea N_2_O fluxes in both downwelling and upwelling seasons. These variabilities are not associated with El Niño-Southern Oscillation (ENSO) indices but are related to interannual differences in upwelling intensity. The time series reveals significant nitrate removal and N_2_O accumulation in both mid and bottom layers, occurring at rates of 1.5 µmol L^−1^ and 2.9 nmol L^−1^ per decade, respectively. Particularly significant is the increase over the past two decades of air-sea N_2_O fluxes at a rate of 2.9 µmol m^−2^ d^−1^ per decade. These observations suggest that changes in the EBUS, such as intensification of upwelling and the prevalence of hypoxic waters may have implications for N_2_O emissions and fixed nitrogen loss, potentially influencing coastal productivity and climate.

## Introduction

Dissolved oxygen (DO) levels below < 89 µM and < 4.4 µM, known as hypoxia and suboxia respectively, impact biogeochemical cycling of bioelements in Eastern Boundary Upwelling Systems (EBUS)^1^. These impacts include changes in inorganic nutrient concentration and GHG production (CO_2_, N_2_O and CH_4_) due to DO levels affecting microbial communities involved in carbon (C), nitrogen (N) and phosphorus (P) cycles^2,3^. N cycling involves microbial transformation of organic and inorganic (DIN: NO_3_^−^, NO_2_^−^and NH_4_^+^) N compounds, including gases such as N_2_ and N_2_O. Microorganisms driving these reactions are sensitive to environmental DO levels, and have crucial roles in determining the rates of key processes as nitrification (aerobic NH_4_^+^ and NO_2_^−^ oxidation), denitrification and anammox (anaerobic ammonium oxidation)^4,5^. Denitrification is the dominant pathway for fixed N loss in the EBUS whereas anammox constitutes a minor fixed N loss pathway^3,6^. Fixed N loss results in the depletion of DIN relative to phosphate altering the ratio of nutrient supply to primary producers^4^.

N_2_O, an important GHG, is produced (accumulated) in the EBUS under variable DO levels. N_2_O primarily results from the reduction of NO_3_^−^ and NO_2_^−^ during partial denitrification^7^. Additionally, aerobic NH_4_^+^ oxidation by bacteria and archaea contributes to N_2_O production^8,9^. Conversely, N_2_O consumption, involving its reduction to N_2_, occurs in anoxic environments^4,10^. The EBUS, characterized by strong DO gradients, facilitates these N transformations under both oxic and suboxic conditions, leading to high N_2_O supersaturation and subsequent outgassing towards the atmosphere^9,11–13^.

DO regulates microbial abundance and activity in N cycling pathways at various temporal scales, including the seasonal^14,15^, interannual e.g. El Niño-Southern Oscillation or ENSO^16^, and decadal variability^17^. Moreover, climate change impacts primary productivity and the strength of the biological carbon pump^18^. It is anticipated to enhance wind-driven upwelling in EBUS, modify El Niño-Southern Oscillation (ENSO) events, and exacerbate ocean deoxygenation and hypoxic events due to factors such as ocean warming, stratification, and acidification^1,19–22^.

While several biogeochemical models project a substantial reduction in DO levels throughout this century, there is a notable scarcity of long-term observational data confirming coastal deoxygenation trends, as evidenced in the Humboldt Current Systems (HCS)^23–25^ and California Current Systems (CalCS)^26^. The consequences of this DO reduction, coupled with intensified upwelling on fixed N loss and the accumulation/depletion of N_2_O have been hypothesized but are still inadequately resolved and may vary^27–31^.

Air-sea N_2_O flux exhibits variability due to changes in solubility, wind intensity, the presence of surfactants on the surface, and N_2_O concentrations in both the atmosphere and the surface ocean^32^. In the ocean, this variability is closely tied to microbial N_2_O production, encompassing processes such as nitrification in the mixed layer and denitrification mainly in Oxygen Minimum Zones (OMZs). Considerations for future climatic scenarios should include alterations in microbial community structure due to ocean warming, lower pH impacting the NH_3_-NH_4_^+^ equilibrium and deoxygenation^4,5,32^.

In the HCS, coastal deoxygenation and water mass distribution changes, due to the intensification of upwelling favorable winds, that lead to OMZs being closer to the surface, are processes of particular interest^25^ As deoxygenation arises from a complex interplay of oceanographic and biogeochemical processes, the superposition of these mechanisms complicates the attribution to any specific driver. The net N_2_O flux across air-sea interface and denitrification contribution appear to be significantly higher under the impacts of climate change on the ocean^30^. This suggests the existence of positive feedbacks between DO changes induced by climate change and microbial N_2_O emission pathways^33^. These feedbacks are substantial enough to account for the observed acceleration in the N_2_O production rate over the next century^9^.

The main question that inspires this research is: What are the implications of observed temporal (seasonal and interannual) variations in oceanographic/biogeochemical parameters, for N_2_O emissions and fixed N loss, in coastal upwelling regions?. The focus of this research is on understanding the oceanographic conditions and specific biogeochemical mechanisms driving N_2_O accumulation and fixed N loss in the upwelling system off central Chile, particularly in relation to hypoxic water dynamics.

## Materials and methods

### Study area

The study area is located on the 40-km-wide continental shelf off central Chile at 36.51° S, 73.13°W, where a strong seasonal coastal upwelling occurs^34^. There, two water masses are present, a surface water mass of relatively fresh (Salinity < 33.8 PSU), oxygenated (> 200 µmol L^−1^ l) and nutrient poor (NO_3_^−^ < 4 µmol L^−1^) water of subantarctic origin (SAAW) and equatorward flowing by the HCS, and a subsurface water mass with high salinity (34.9 PSU) oxygen-depleted (4.4—44 µmol L^−1^) and nutrient-rich (20 < NO_3_^−^ < 40 µmol L^−1^) corresponding to equatorial subsurface water (ESSW) and flowing poleward by the Peru Chile undercurrent^35^. When winds favorable to coastal upwelling stress the ocean surface, the SAAW is displaced westward and the ESSW shoals and even reaches the surface^25,36^.

The sampling station (time series station or TSS 18) is located at 92 m depth isobath at 18 nm from the coast (Fig. Supplementary 1) and has been sampled monthly since 2002 and quarterly from 1997 to 2002 as part of time series study of Concepcion University (Chile), constituting one of the few existing long-term monitoring programs with monthly or quarterly sampling cruises^25,37–39^. Our measurements and estimations cover a period of over more than two decades for physical variables (1997–2023) and nutrients, DO and GHGs including N_2_O (2002–2023).

### Sampling and analytical methods

Seawater samples were collected with Niskin bottles from various depths and samples were used to determine nutrient and GHG concentrations. This dataset was already partially analyzed to elucidate DO variability over the past 20 years^25^. Sampling and analytical methods related to N_2_O, and nutrients are described by Farías et al^12^. Calibration procedures, error and uncertainty of N_2_O measurements are presented in Wilson et al^40^.; for nutrient (NO_3_^−^, NO_2_- and HPO_3_^−^) filtered seawater was collected during the whole sampling period and analyzed using manual (1997–2007) and automatic (2008-present) colorimetric methods according to Grasshoff et al.^41^(more information see^12^); whereas NH_4_^+^ was determined without filtration by fluorometric method^42^.

### Data analyses

DIN was estimated as the sum of NO_3_^−^, NO_2_^−^, NH_4_^+^ (Eq. 1). The latter was analyzed during the first 10 years, thus monthly NH_4_^+^ time series data by depth was complemented with climatological values when NH_4_^+^data was missing to estimate DIN. The proportion of NH_4_^+^ relative to the other N inorganic forms is very small and has almost no effect on DIN where the main driver is NO_3_^−^.

1 $$N_{DIN} = \left[ {{\text{NO}}_{{3}}^{ - } } \right] \, + \, \left[ {{\text{NO}}_{{2}}^{ - } } \right] \, + \, \left[ {{\text{NH}}_{{4}}^{ + } } \right]$$

N deficit (Eq. 2) and N* (Eq. 3) proposed by Broecker & Peng (1982) and Gruber and Sarmiento (1997)^43,44^, respectively, was estimated as:

2 $${\text{Ndeficit}} = N_{DIN} - { 16}\left[ {{\text{PO}}_{{4}}^{{{3} - }} } \right]$$

3 $${\text{N}}* = \, 0.{87 }\left\{ {{\text{N}}_{{{\text{DIN}}}} - { 16}\left[ {{\text{PO}}_{{4}}^{{{3} - }} } \right] \, + {2}.{9}} \right\}$$

air-sea N_2_O fluxes were estimated according to:

4 $$F = kw_{(T^\circ , \, salinity)} \left[ {{\text{N}}_{{2}} {\text{O}}} \right]_{{\text{w}}} - \, \left[ {{\text{N}}_{{2}} {\text{O}}} \right]_{{{\text{eq}}}}$$

where *kw* is the transfer velocity from the surface water to the atmosphere, as a function of wind speed, temperature, and salinity from the mixed layer depth (MLD), *Cw* is the mean N_2_O concentration in the MLD and *C*eq is the gas concentration in the MLD expected to be in equilibrium with the atmosphere, according to Weis and Price^45^. Transfer gas velocity *Kw* as a function of wind speed was based on Wanninkhof (2014) or W2014^46^ and compared with that of Wanninkhof (1992) or W92^47^ (see Supplementary methods).

Climatologies were calculated by the Fast Fourier transform method fitting the seasonal frequency harmonics for each variable obtained in the TSS. Then the climatology was subtracted from the time series to obtain the anomaly. To quantify the proportion of hypoxic waters, present at the sampling location the proportion of the sampled water column under a certain threshold was computed as the total sampling depth minus the shallowest register of DO < threshold. Four DO thresholds were defined to then find the best correlation with N_2_O concentrations, these were 89, 22, 11 and 4.4 µmol L^−1^.

Oceanic and coastal episodes of El Niño (EN) and La Niña (LN) were detected using two indexes, the Oceanic Niño Index (ONI) and the coastal El Niño index (ICEN) (see Supplementary methods). Some outliers were removed from physical and nutrient data, particularly from the start of the time series where anomalous values were found. In addition, to assess variability among years, the dataset from 2002 to 2023 was divided into 21 seasons, from September to March (upwelling favorable season) and from April to August (non-upwelling season). Cumulative alongshore (south–north) wind stress for each season was obtained from the cumulative sum of wind stress from the start to finish of each cycle. As in de la Maza and Farias^25^, wind stress was calculated according to Nelson et al.^48^ for u (cross-shore) and v (alongshore) components using wind speed data from Carriel Sur weather station and ERA5 from the European Center for Medium-Range Weather Forecasts (see Supplementary methods).

## Results and discussion

### Seasonal and interannual variability in nutrient and N_2_O concentrations, N deficit and N_2_O fluxes

The EBUS located in the Pacific Ocean exhibit distinct temporal regimes based on latitude and proximity to the equatorial band, in which they are sited. The EBUS in mid latitudes (30°–45°S) are characterized by a strong seasonality, primarily influenced by the latitudinal migration of high pressure centers (anticyclones) that drive their wind regimes^34,49^. Off central Chile, the South Pacific anticyclone (SPA) drives local winds along the coast and shifts the vertical distribution of the SAAW and ESSW. In the austral fall-winter (April-August), the SPA undergoes a northward migration, giving rise to frequent midlatitude cyclones. This leads to weaker or even northerly winds and a coastal downwelling. Conversely, during spring–summer (September to March), the SPA's southward displacement induces prevailing equatorward alongshore winds, fostering coastal upwelling^50^. This upwelling laterally and vertically transports the ESSW over the continental shelf, where our long TSS is situated (Fig. Supplementary 1), fostering high rates of primary productivity^51^ and modulating DO variability and air-sea N_2_O fluxes^12,25^. Table 1 presents basic statistics of all variables and parameter estimates for the 2002–2023 TSS; for most of the variables and estimates, the median and average estimates are very close values, except for N_2_O level and its air-sea flux. This discrepancy reveals numerous extreme values, referred to as N_2_O hot moments, where N_2_O values exceeded 2 standard deviation units^12^.

**Table 1:** Descriptive basic statistics for physical and biogeochemical variables throughout the water column.

Variable	Mean	Median	Sd	Se	Min	Max	n
Temperature (°C)	11.69	11.67	0.91	0.00	9.14	18.07	2048
Salinity (PSU)	34.28	34.42	0.39	0.00	20.24	35.93	2030
DO (µmol L^−1^)	123.42	121.54	96.85	2.13	0.00	373.06	2074
NO_3_^−^ (µmol L^−1^)	17.35	17.83	8.22	0.20	0.00	37.85	1730
NO_2_^−^ (µmol L^−1^)	0.60	0.35	1.01	0.02	0.00	9.74	1737
HPO_4_^3−^ (µmol L^−1^)	1.96	2.03	0.76	0.02	0.06	3.62	1730
NH_4_^+^ (µmol L^−1^)	0.50	0.20	0.74	0.02	0.00	4.8	925
N_2_O (nmol L^−1^)	30.76	23.25	31.33	0.77	2.34	492.14	1654
Inorganic N:P ratio	9.45	9.22	5.38	0.13	0.12	42.14	1707
N* (µmol L^−1^)	− 10.41	− 9.71	8.44	0.20	− 42.41	28.50	1707
N deficit (µmol L^−1^)	− 13.20	− 12.57	8.29	0.20	− 45.31	25.60	1707
N_2_O flux (µmol m^−2^ day^−1^)	9.54	3.21	20.15	1.35	− 6.48	215.39	223
N_2_O flux (µmol m^−2^ day^−1^)*	6.03	3.03	7.81	0.54	− 6.48	39.92	212
N_2_O flux (µmol m^−2^ day^−1^)**	18.59	6.09	39.77	2.60	− 6.37	415.71	234

Air-sea N_2_O flux without asterisk calculated using Carriel Sur wind speeds; *N_2_O flux with N_2_O hot-moments excluded for calculations using weather station data. Lastly, ** are N_2_O fluxes calculated using ERA5 wind speeds.

#### N species content and N deficit

**Figure 1:**
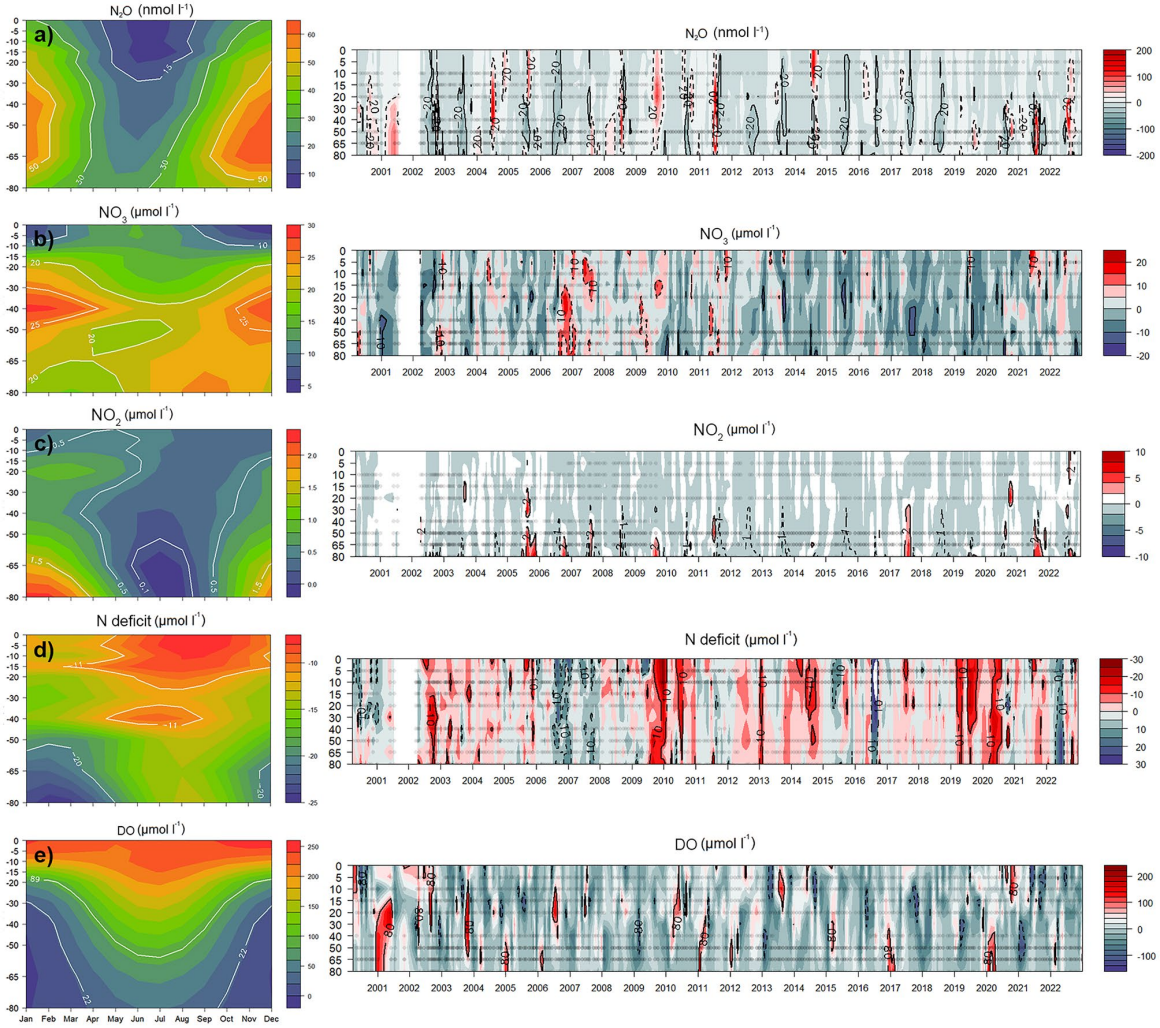
Annual cycles (left) and temporal variability of climatological anomaly in the water column (right) of (**a**) N_2_O (nmol L^−1^), (**b**) NO_3_^−^ (µmol L^−1^) (**c**) NO_2_^−^ (µmol L^−1^), (**d**) N deficit (µmol L^−1^) and (**e**) dissolved oxygen (µmol L^−1^) during the study period (August 2000–2023) at TSS 18.

Temporal variability of N_2_O concentrations reveal a marked seasonality (Fig. 1a, left panel) with high interannual differences (Fig. 1a, right panel), ranging from 2.34 to 492 nmol L^−1^ along the time series (mean ± SD = 30.76 ± 31.33 nmol L^−1^). Low N_2_O levels occur in fall-winter and increase in spring–summer. The highest values consistently occur under hypoxic conditions of 22 < DO < 11 μmol L^−1^, the threshold that correlates most strongly with N_2_O in the MLD during the upwelling period (Fig. 1a and e). N_2_O accumulations contrast with low N_2_O levels in bottom water close to the sediments during late summer, occasionally reaching sub-saturated levels around 40% (Fig. 1a).

A similar accumulation/depletion pattern is observed for NO_3_^−^ (Fig. 1b), exhibiting a clear seasonality which is opposite to DO. Lower NO_3_^−^ levels are associated with oxygenated waters, while higher levels are linked to hypoxic/suboxic waters during downwelling and upwelling seasons, respectively. This cycle aligns with the seasonal influence of the ESSW demarcated by the 26.2 isopycnal^25^. The bottom waters during late summer (January and March) exhibit a noteworthy NO_3_^−^ depletion, with levels lower than expected for the ESSW. This suggests a substantial NO_3_^−^ consumption under suboxic conditions, occurring either in the bottom water or in the sediments^52^. This corresponds with the N_2_O depletion (Fig. 1a), indicating that canonical denitrification (the complete and sequential reduction of NO_3_^−^ to N_2_) occurs^4^. NO_2_^−^ distribution over time generally remains below 0.2 µmol L^−1^ throughout the water column, except near the sediments where higher levels (up to 9.73 µmol L^−1^) are observed, particularly in summer (Fig. 1c), coinciding with suboxic conditions (Fig. 1e). NH_4_^+^ shows a similar distribution as NO_2_^−^ (data not shown).

**Figure 2:**
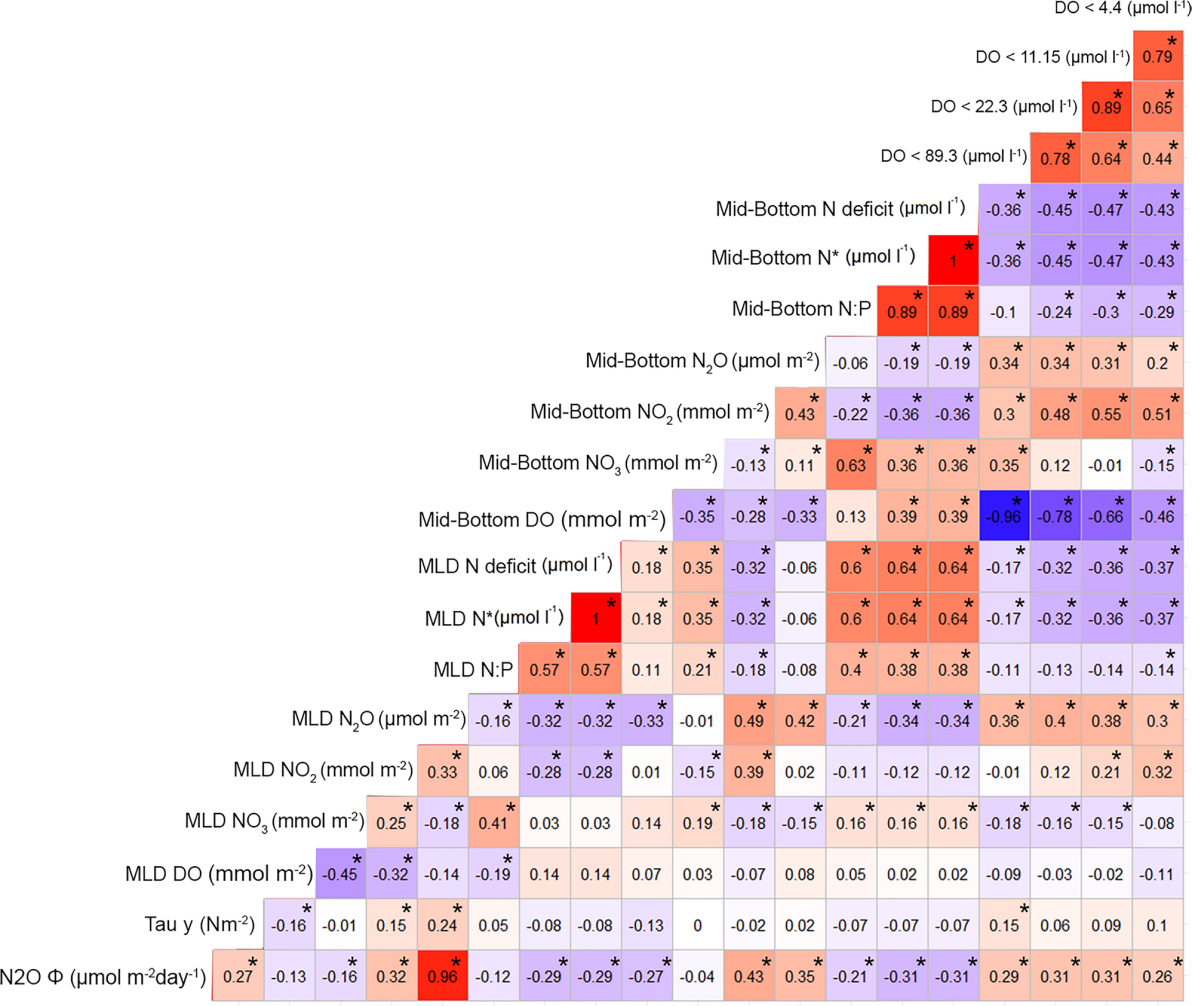
Heatmap of correlation matrix among biogeochemical variables that include surface and mid-bottom inventories of N_2_O, NO_3_, NO_2_, DO, as well as estimates of N deficit, N:P, N*, N_2_O fluxes, alongshore wind stress and Hypoxic Volumes at 4.4, 11.15, 22.3 and 89.3 (µmol L^−1^) thresholds. Asterisk represents statistical significance.

The inorganic nutrient N∶P ratios consistently fall below than the expected Redfield ratio^53^ in both surface (9.04 ± 1.85) and subsurface (10.15 ± 2.74) layers, whereas N deficit, ranging from 25.60 to − 45.3, increases with depth (Fig. 1d). The maximum N deficits up to − 45.3 µmol L^−1^ (mean ± SD: -13.20 ± 8.29) are primarily influenced by the lateral and vertical advection of denitrified water (ESSW) poleward transported by the Peru–Chile undercurrent^35^. This is coupled with local dissimilative NO_3_^−^ reduction during anaerobic organic matter mineralization in bottom waters and sediments. Also, N* values show a similar range (mean ± SD: -10.41 ± 8.44 µmol L^−1^), indicating that denitrification predominates over N fixation^44^. As expected, N deficit strongly and negatively correlates with NO_3_^−^ content and the volume of hypoxic waters at various threshold levels (Fig. 2). Notably, the highest correlations between these parameters are identified with DO levels around 11 and 22 µmol L^−1^ (from 50- 80 m depth).

#### N_2_O exchanges across the air-sea interface

**Figure 3:**
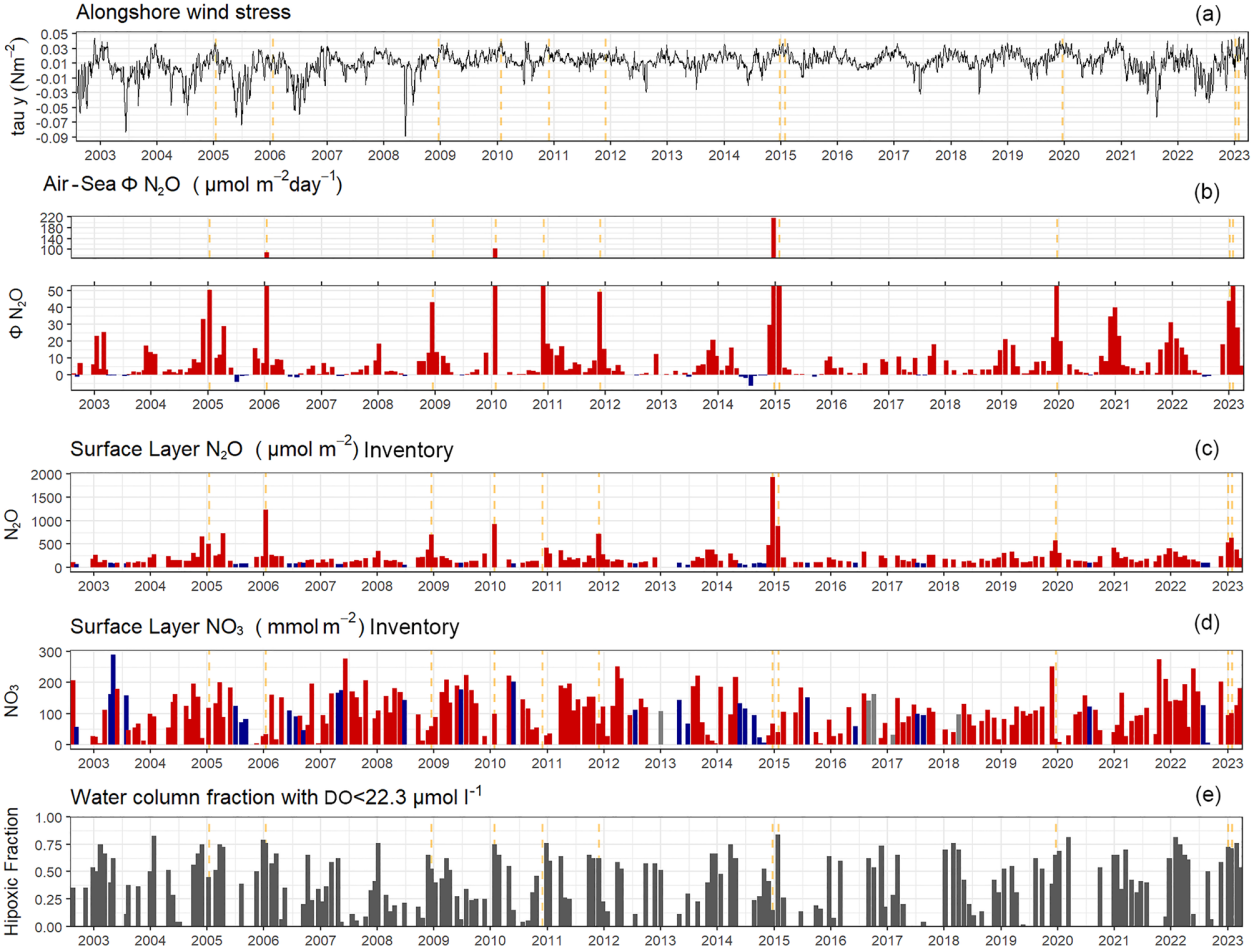
Time series of (**a**) alongshore wind stress (N m^−2^), (**b**) estimated air–sea N_2_O fluxes (μmol m^−2^ d^−1^) (**c**) N_2_O µmol m^−2^ and (**d**) NO_3_^−^ (mmol m^−2^) inventories in surface layer (0–10 m depth) and (**e**) Hypoxic proportion (DO < 22.3 µmol l^−1^) during 2000 to 2023 at TSS18. Vertical dashed lines indicate the dates at which the N_2_O hot moments were present.

The EBUS represent significant sources of N_2_O to the atmosphere, and their emission rates exhibit spatial and temporal variations influenced by specific atmospheric and oceanographic conditions^54,55^. A comprehensive understanding of this variability is crucial for precise regional and global assessments of N_2_O emissions. Figure 3 presents a time series of (a) wind stress (b) air-sea N_2_O fluxes using Carriel Sur weather station winds (c) N_2_O and (d) NO_3_^−^ inventories in the MLD and (e) hypoxic water volume less than 22 µmol L^−1^. Air-sea N_2_O fluxes show a strong seasonal variation, ranging from -6.48 to 215.3 μmol m^−2^ d^−1^ (mean ± SD: 9.53 ± 20.15), characterized by substantial outgassing during the upwelling season and, conversely, lower or even N_2_O influx (from atmosphere to surface ocean) during downwelling season. This N_2_O sink in wintertime is a very conspicuous regional feature associated with the surface SAAW or modified SAAW (25.25–25.75 kg m^−3^) that present N_2_O sub-saturations in adjacent oceanic waters; it appears to be due to a biological uptake (microbial N_2_O fixation) rather than physical process (such as water mixing or changes of T°C or S)^56^. It should be noted that the average air-sea N_2_O flux estimated from 2002 to 2023 is lower than those previously reported from 2002 to 2013, using the W92 parametrization and long-term wind stress^12^. However, considering the existing synoptic wind fluctuations in the study area, such as relaxed and active upwelling events lasting between 2 and 7 days^57^, the average wind speed of the seven days prior to observations is considered the most reliable estimate for air-sea N_2_O fluxes in the study area. When estimating N_2_O fluxes with ERA5 wind datasets, the values range from -7.21 to 416 μmol m^−2^ d^−1^ (mean ± SD: 19.11 ± 39.91). A comparison of N_2_O fluxes obtained by different wind speed datasets, reveals that reanalysis data tends to produce air-sea N_2_O flux estimates that exceed those of the Carriel Sur weather station by about 49%. It is important to note that the meteorological station at Carrier Sur airport has a continental location (close to the coast), implying a retarding or frictional force on the wind that could impact on wind strength. Results reported by Wong et al.^58^, who conducted an ERA-5 Wind Data validation in the study area, suggest that ERA5 can reproduce the weather conditions at Carriel Sur weather station (r2 = 0.58) with a small bias (0.72 m s^−1^). It is noteworthy that air-sea gas flux estimates scale exponentially with wind speed^46^), indicating that slightly higher wind speeds offshore may result in significantly higher gas flux rates. By comparing the continental measurements of Carriel sur and the offshore pixel of ERA-5 this dependence is considered providing a range of estimates, Carriel sur being the most conservative of the two.

Much temporal variability in air-sea N_2_O fluxes is due to the presence of N_2_O flux hot moments, characterized by disproportionately high N_2_O emission exclusively during the summertime (Fig. 3b). Hot moments are not necessarily associated with the highest upwelling favorable winds (Fig. 3a) but correspond to high surface N_2_O inventories (Fig. 3c) but low for NO_3_^−^ (Fig. 3d); the latter may be an indication of a strong consumption by phytoplanktonic assimilation. In addition, hot moments, previously described in the TSS^12^, match with N_2_O accumulations in 15–50 m depth and high chlorophyll-a levels. This suggests that an intense microbial activity accompanies the development of such accumulations, and that part of the N_2_O exchanged with the atmosphere may come from the surface. This surface N_2_O is likely produced by nitrifying bacteria coupled with high rates of particulate organic matter accumulation and NH_4_^+^ regeneration^8^. A significant proportion of N_2_O originates from the mid-bottom layer, which is identified as the primary source of N_2_O exchanges with the atmosphere (see below). This layer is characterized by DO concentrations below 22 µmol L^−1^. However, it is important to note that the hot moments do not consistently coincide with the periods of maximum hypoxic volumes (Fig. 3e).

The occurrence of these events, totaling 11 in number, introduces significant variability in air-sea N2O flux. Specifically, when excluding these 'hot moments,' mean N2O fluxes decrease by 33%. We believe that the presence of N_2_O hot moments may be more frequent than currently observed, especially if continuous observation of the surface ocean were maintained. This underscores the importance of synoptic and high-frequency variability^12^ and emphasizes the need to consider such factors when estimating regional N_2_O fluxes associated with the EBUS the EBUS.

Strong positive correlations are found among N_2_O fluxes, N_2_O contents at mid-bottom (30–80 m depth) layers, wind stress and the proportion of hypoxia water at various levels (< 89, 22, 11 and 4.4 µmol L^−1^) (Fig. 2). The N_2_O content exhibits the strongest correlation when 4 < DO < 89 μmol L^−1^, indicating that the hypoxic range plays a significant role in controlling N_2_O levels in the water column. N_2_O flux also correlates with the increase in the N deficit and the accumulation of NO_2_^−^ (Fig. 2), indicating that N_2_O accumulation and its subsequent exchange primarily proceeds via partial denitrification under hypoxic levels (< 4 µmol L^−1^). The differential sensitivity of N_2_O, NO_3_- and NO_2_^−^, to hypoxic DO levels is depicted in Fig. Supplementary 2. Recent research, based on experimental data, has revealed that the dominant N_2_O source in oxygen deficient waters is NO_3_^−^ reduction where rates of NO_3_^−^ reduction are found to be one to two orders of magnitude higher than those of NH_4_^+^ oxidation^9,10^. Given the current alterations in intensity and timing of wind within EBUS, which vary widely according to region^59^; changes in upwelling dynamics may influence the proportion of hypoxic/suboxic waters^25^ and therefore the N_2_O balance between production and its consumption. However, N_2_O cycling rates in suboxic regions appear to be an order of magnitude higher than predicted by current models^30^. The rapid rate of N_2_O cycling coupled to an expected expansion of OMZs imply future increases in N_2_O emissions, representing positive feedback of the global marine N_2_O sources.

### Variability among annual cycles of biogeochemical variables and drivers

Long time series studies enable us to discern the influence of low frequency climate processes such as the ENSO or the Pacific Decadal Oscillation (PDO) on physical and biogeochemical processes in the EBUS. The ENSO acts as a significant remote forcing influencing the HCS through atmospheric teleconnections and ocean currents, including poleward propagating coastal Kelvin waves^60,61^. ENSO impacts various oceanic parameters such as sea surface temperature (SST), sea level, thermocline depth, surface and subsurface current flows, and upper ocean properties. In terms of biogeochemical processes, the ENSO is expected to induce changes in nutrient availability (N deficit) and primary productivity as thermocline deepens and the wind weakens^62^. But the responses in physical and biogeochemical properties differ markedly between the ENSO events and they even have different responses along the latitudinal gradient as those reported along the CalCS and the HCS^23,63–66^.

At low latitudes, EBUS display a significant sensitivity of N_2_O content and air-sea N_2_O flux in response to ENSO^9,13,16^. In literature, surface N_2_O supersaturation in the shelf area during the 2015 El Niño was observed to be nearly an order of magnitude lower than non-El Niño years, implying a significant reduction in air-sea N_2_O efflux (75–95%)^9^. The coupling between physics and biogeochemistry varies between strong and moderate El Niño events^23,67,68^. During strong El Niño events such as the one that occurred in 1997–1998, Kelvin-wave-induced downwelling conditions switched off upwelling, drastically reducing nutrient availability and increasing oxygenation^67^. In contrast, during moderate and weak El Niño events observed in the post-2000 period, equatorial Kelvin wave activity is relatively weaker^69^, maintaining mean upwelling conditions and producing smaller anomalies in nutrient and DO levels^25,70^.

The study period coincides with three notably recorded strong Pacific EN events as 1997–98, 2015–2016 (Godzilla) and 2017 (coastal EN), which rank among the ten strongest EN events recorded in the last century^71^, as well as other moderate and weak EN events in conjunction with LN events (Fig. Supplementary 3 and Table S1). However, non-significant correlations are identified among central (ONI) and eastern Pacific (ICEN) ENSO indexes and biogeochemical variables when all data are considered (Fig. Supplementary 4). Only weak correlations are found with surface T°C, salinity and DO, even though a prolonged oxygenation event occurred in 1997–1998^25^. There is also no relationship between the ENSO indices with the wind stress or with the N_2_O fluxes, suggesting that at mid-latitudes, biogeochemical properties do not exhibit a noticeable response. In the eastern tropical Pacific (ETP) off Peru, significant anomalies in oceanographic and biogeochemical parameters occur in association with the ENSO due to its proximity to the equatorial Pacific. There, ENSO induces changes in ocean circulation patterns, upwelling strength, and thermocline depth, leading to vertical water mass redistributions^72,73^. These dynamics affect the thickness and extent of equatorial subsurface water (ESSW), which is closely linked to the OMZ underlying Tropical Surface Water (TSW)^74,75^. Fluctuations in the extent of the OMZ in this region result from changes in DO supply primarily from lateral (zonal) margins^36,76^. These shifts in the distribution of oxygen-poor waters may have significant implications for the biogeochemical N cycle, particularly in relation to N_2_O emissions^77^. At mid-latitudes off central Chile, a different distribution of water masses is observed respect to tropical regions, with ESSW moving southwards along the Peru–Chile countercurrent, surrounded above and below by well-oxygenated waters such as Subantarctic SAAW and Antarctic Intermediate Water (AIAW). The dominant meridional transport in mid-latitudes, along with the different distribution of water masses, may explain the lack of correlation between biogeochemical parameters and ENSO.

**Figure 4:**
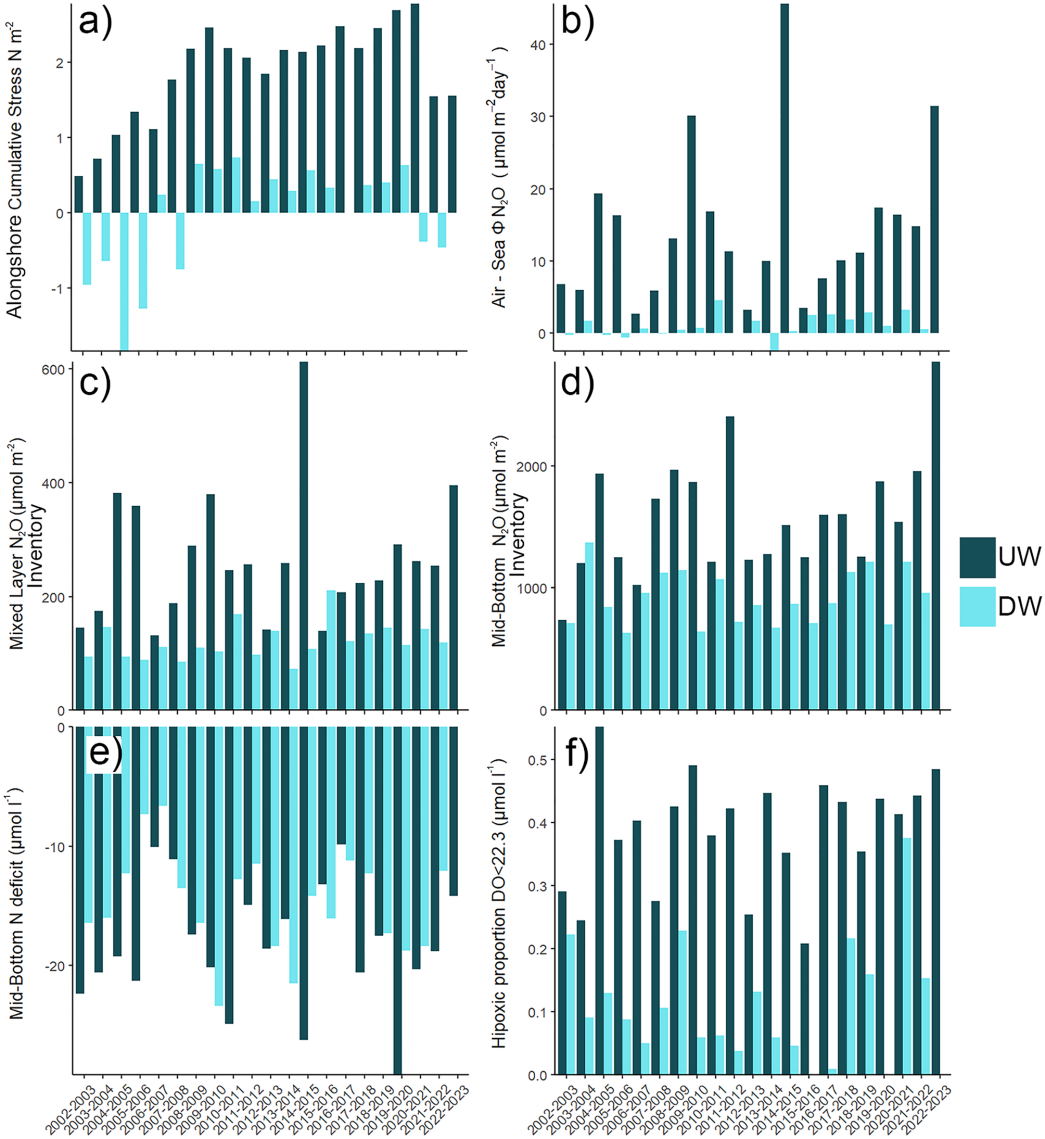
Annual variability among cycles including non-upwelling (april-august) and upwelling (September to March) season of cumulative wind stress (**b**) air sea N_2_O fluxes (**c**) monthly averages of surface (**d**) N_2_O mid-bottom layer inventories (**e**) N deficit and Hypoxic proportion (DO < 22.3 µmol l^−1^); 2000–2023 at TSS18. Uw and Dw mean upwelling and downwelling seasons, respectively.

To disentangle interannual variability, we separate and compare 21 upwelling and downwelling seasons from the beginning of the upwelling season (Sept.) to the end of downwelling (Aug.). To synthesize information for each season and annual cycle, cumulative alongshore wind stress along with nutrient and N_2_O inventories by the surface and mid-bottom layers are shown in Fig. 4 and Table S1. Strong variations in N_2_O fluxes are observed across annual cycles, as well as N_2_O inventories and N deficit, which are the variables with highest correlations with N_2_O fluxes on interannual scales (Fig. Supplementary 6, UW row). The multivariate relationship between N_2_O flux variability and other variables is further explored with a PCA where principal Component 1 (PC1) involves an inverse relationship between mid-bottom N deficit and ONI index with the other variables. This aligns with the expected for upwelling induced variability. PC1 accounts for a substantial proportion of the total variance with 33.7% for downwelling, 41.8% upwelling and 57.8% when both cycles are considered.

Variance distributions for each variable within PC1 are provided in Table 2. PC1 encompasses over 80% and above 70% of air-sea N_2_O flux and surface N_2_O inventory variability, respectively. Differences between upwelling and downwelling seasons emerge in the variance of hypoxic proportion and N deficit within the first component. These N_2_O fluxes are approximately 6–7 times higher during upwelling seasons compared to downwelling seasons (Fig. 4b). Such distinctions between seasons collectively contribute to the observed fluctuations in aggregated seasons, encompassing both inter-seasonal variations and interannual variability. The interannual differences between upwelling seasons are driven by the variability of N_2_O levels in MLD and its air-sea fluxes, hypoxic proportion and mid-bottom N_2_O inventory, with a small incidence of cumulative wind stress and N deficit, while ENSO (ONI) is almost uncorrelated. For downwelling seasons, the differences lie in N_2_O fluxes, N_2_O levels in the MLD and Mid Bottom layers. Counterintuitively, year to year differences in wind stress during downwelling season are more relevant than in the upwelling season, where due to the combined effect of upwelling and enhanced exchange due to wind intensity, a greater ponderation of this variable was expected.

**Table 2:** Percentage of variance contained within the first covariance pattern (PC1) for each original variable.

Dominant PC	Upwelling season	Downwelling season	Aggregated cycles
N_2_O flux	81.2	85.9	84.5
DO < 22.3 (µmol l^−1^)	49.5	6.7	74.2
N deficit	15.3	2.4	29.1
Mixed layer N_2_O	81.0	74.8	83.2
Mid-bottom N_2_O	47.4	41.2	67.7
Cumulative alongshore wind stress	14.2	21.5	64.7
ONI	4.0	2.0	0.4

These findings suggest notable interannual variations in air-sea N_2_O fluxes, with distinct controlling factors for each season. During the spring–summer or upwelling season, the highest air-sea N_2_O fluxes are estimated, and variations in the intensity and frequency of upwelling events significantly influence these observed differences. In contrast, during autumn–winter, the heightened presence of the oxygenated SAAW (respect to the ESSW) along with downwelling processes, serves to curtail the N_2_O exchange and production of N_2_O. Other biogeochemical processes should be underlying, particularly the preponderance of reductive processes (denitrification) that dominate in spring and summer, in contrast to oxidative processes (nitrification) that prevail in fall-winter. In fact, during the downwelling season, a greater diversity of activity nitrifying archaea and higher nitrification rates have been recorded in the area affecting N recycling^78,79^.

### Interannual trends in nutrients (N deficit), N_2_O content and its air -sea fluxes

Temporal trends of biogeochemical variables and indices estimated for different layers (the MLD: 0-10 mm, subsurface: 11–29 m where oxyline and pycnocline are located, mid 30-65 m, and bottom 66–80 m) are presented in Table 3. The NO_3_^−^ trend indicates that this nutrient is removed in mid and bottom layers at significant rates about 0.15 µmol L^−1^ y^−1^, where DO shows the highest consumption rates (Table 3). This rate corresponds to a NO_3_^−^ loss per decade of 1.5 µmol L^−1^, a value that can be significant for the ecosystem if the fixed N loss persists for the next decades^80^. This NO_3_^−^ reduction corresponds to N deficit and N* indices, which increase similarly, indicating that NO_3_^−^ and NO_2_^−^ is being reduced to N_2_O/N_2_. On the other hand, in the same layers, an accumulation of NO_2_^−^ at rates ranging from 0.11 to 0.22 µmol L^−1^ decade^−1^ is estimated, only 7% of the NO_3_^−^ reduction. This small NO_2_^−^ accumulation indicates that NO_2_^−^ continue to be reduced or oxidized through processes already described in the study area such as dissimilative NO_2_^−^ reduction, aerobic NO_2_^−^ oxidation (nitrification), anaerobic NH_4_^+^ oxidation (anammox) and even nitrifier denitrification, where NO_2_^−^ is used as electron acceptor or donor^81,82^. Conversely, N_2_O, an intermediate of denitrification or a by-product of nitrification, accumulates in the mid layer at rates of 0.14 nmol L^−1^ y^−1^, but it is consumed in the bottom layer (− 0.07 nmol L^−1^ y^−1^) (Table 3). These trends patterns are expected based on the DO ranges observed in each layer and the differential sensitivity of the enzymes involved in the sequential reduction of NO_3_^−^ to N_2_^5^. These intricate observed patterns underscore the complex interactions between DO availability and N cycling processes, emphasizing the influence of seasonal and interannual variability on the biogeochemical processes in the EBUS. The findings shed light on the mechanisms underlying N_2_O dynamics and highlight the significance of DO levels in governing N transformations in the marine ecosystems.

**Table 3:** Interannual trends of physical and biogeochemical variables and estimates at the bottom (65–80 m), mid (30–50 m), subsurface (15–20 m) and surface (0–10 m) layers.

Variable	Layer	Unit\year	Intercept	n
DO (μmol L^−1^)	Bottom (65–80)	− 0.264	0.281	224
	Mid (30–65)	− 1.016*	0.962	231
	Surface (0–10)	− 0.691	0.661	242
N_2_O (nmol L^−1^)	Bottom (65–80)	0.028	− 0.990	150
	Mid (30–65)	0.295	− 12.596	187
	Surface (0–10)	0.148	− 6.419	224
NH_4_^+^ (μmol L^−1^)	Bottom (65–80)	0.000	0.005	93
	Mid (30–65)	− 0.007	0.301	106
	Surface (0–10)	− 0.006	0.245	117
NO_2_^−^ (μmol L^−1^)	Bottom (65–80)	0.011	− 0.466	186
	Mid (30–65)	0.005	− 0.228	190
	Surface (0–10)	0.010*	− 0.407	224
NO_3_^−^ (μmol L^−1^)	Bottom (65–80)	− 0.138*	5.725	188
	Mid (30–65)	− 0.058	1.947	190
	Surface (0–10)	0.065	− 2.667	224
Salinity (PSU)	Bottom (65–80)	− 0.003	0.160	223
	Mid (30–65)	0.000	0.024	221
	Surface (0–10)	0.011	− 0.450	236
Temperature (°C)	Bottom (65–80)	− 0.003	0.100	226
	Mid (30–65)	− 0.006	0.254	224
	Surface (0–10)	− 0.007*	0.685	239
N* (μmol L^−1^)	Bottom (65–80)	− 0.15	− 9.36	186
	Mid (30–65)	− 0.16*	− 5.13	190
	Surface (0–10)	− 1.10	− 3.26	224
N deficit (μmol L^−1^)	Bottom (65–80)	− 0.15	− 11.85	186
	Mid (30–65)	− 0.20*	− 6.39	190
	Surface (0–10)	− 0.10	− 6.09	224
N_2_O Flux (μmol m^−2^ day^−1^)	Air–sea interface	0.29	− 2.97	223
N_2_O Flux (μmol m^−2^ day^−1^)**		0.22*	− 3.69	212
N_2_O Flux (μmol m^−2^ day^−1^) using ERA5		0,16	12,42	223

Statistics include slope and intercept and statistical significance (linear regression). Units indicate changes per year.

*Statistically significant; **estimated N_2_O flux with hot moment removal.

The reduction of NO_3_^−^ concentration and the increase of N deficit in more than two decades could indeed have significant consequences on primary productivity and the composition of the phytoplankton community, subsequently impacting marine food webs. The decrease in the inorganic N* and N deficit could lead to N limitation in the system, affecting nutrient recycling processes such as the POM remineralization and variations in the N:P ratio of POM^83^; these changes could further impact nutrient availability, overall productivity and phytoplankton composition, favoring certain phytoplanktonic species better adapted to these altered nutrient proportions^84^, and/or displaying environmental and physiological acclimation responses (e.g., cellular macronutrient contents^80,85^).

Wind driven upwelling intensifications off Chile seem to induce contrasting trends in oceanographic conditions, phytoplankton biomass and primary productivity with different or even opposing rates of change in different areas along the Chilean coast; To the north of 30° S, increases in upwelling winds, decreased sea surface temperature (SST), and enhanced chlorophyll-a concentration are observed in the nearshore areas, likely influenced by heightened land-sea pressure gradients (known as the Bakun's effect). However, to the south, the dynamics of the South Pacific Anticyclone (SPA) appear to dominate over the spatiotemporal fluctuations of chlorophyll^86,87^.

The observed long-term increase in hypoxic and suboxic water volumes is primarily controlled by enhanced alongshore wind stress^25^. This influences the vertical distribution of SAAW and ESSW (i.e. increasing the proportion of the ESSW to the SAAW on the continental shelf) by having more frequent presence of hypoxic (N_2_O rich) waters near the surface^25,33^. While dissimilatory NO_3_^−^ reduction leads to a decrease in DIN, the release of bioavailable phosphorus (P) from anoxic sediments may contribute to an increase in P availability in coastal waters^88^. When the inorganic N deficit takes negative values, indicating an excess of P relative to nitrogen (i.e. N:P lower than the Redfield ratio), phytoplankton growth may be limited by inorganic N availability. This imbalance could lead to shifts in the composition of phytoplankton and bacterioplankton communities, potentially impacting marine productivity^1,21,88^.

Finally, the trend of air-sea N_2_O flux shows an increase over the years at rates 0.29 µmol m^−2^ d^−1^ y^−1^ (p: 0.18) (Table 3); when hot moments are removed, a significant f N_2_O flux trend of 0.22 µmol m^−2^ day^−1^ y^−1^, lower by a 24% , is estimated. Since the air-sea gas flux is a function of the wind and the gas concentration gradient between the MLD and the atmosphere, both factors control the gas exchange; but in an upwelling area, the gas concentration in the MLD strongly depends on accumulation (production) of N_2_O into mid-bottom water, which is then vertically advected to surface by favorable upwelling wind stress.

If we consider that the average N_2_O flux (Table 1) is representative of a small continental shelf off central Chile delimited by isobath of 200 m (about 41,105 km^2^), this coastal area significantly contributes to global marine N_2_O emission with about 6.3–12.6 Gg N_2_O-N per year (depending on used wind data). However, the most relevant finding is that in 20 years, the area has increased its N_2_O emission by 1.7%, a much higher rate than that reported for the global ocean^89^. Moreover, if we take into consideration that N_2_O hot moments may occur more frequently than observed during this study’s sampling cycle (at intervals of 30 days or more), the N_2_O emission rate could be higher.

### Conclusions and perspectives

Strong positive correlations found between N_2_O contents at mid layers along with the proportion of hypoxic water (4 < DO < 89 µmol L^−1^), indicate that this hypoxic range plays a significant role in controlling N_2_O levels mainly via partial denitrification. Given the accelerated deoxygenation observed in the EBUS such as central Chile^25^, the accumulation of N_2_O could be greater in the next decades.

In coastal upwelling areas extending onto the continental shelf, hypoxic conditions often prevail in the mid-waters, fostering conditions conducive to incomplete denitrification and subsequent N_2_O production. However, if DO levels decrease sufficiently (< 4 µmol L^−1^) and expand, dissimilative N_2_O consumption can occur through strictly anaerobic microbial processes^83^. Therefore, improved observational data and biogeochemical models are imperative to capture the variability in N_2_O production/consumption rates in the mid and long term^90^.

Despite ENSO being the main driver of interannual variability in the HCS, no significant correlations were found between ICEN and ONI indices with air-sea N_2_O flux. However, there is high variability among years, driven mostly by differences in cumulative wind stress, the presence of upwelled hypoxic waters with high N_2_O concentrations near the surface and the occurrence of N_2_O hot moments. These differences are exacerbated in the upwelling season as the N_2_O air-sea fluxes are up to 7 times higher and hot moments are observed exclusively during this period of the year.

Our findings reveal an increase in air-sea N_2_O flux over the study period, with rates of 0.29 µmol m^−2^ d^−1^ y^−1^. Furthermore, the N_2_O exchange rate is notably influenced by the existence of hot moments, indicating an accumulation of N_2_O in surface waters. This accumulation is strongly correlated with favorable upwelling wind stress. Therefore, understanding the potential increase in marine N_2_O emissions observed in upwelling systems, which implies positive climate feedback, is pivotal for developing effective strategies to mitigate its influence on the Earth's atmosphere. Predictions regarding coastal upwelling towards the end of this century remain uncertain, given the intricate and competing changes in upwelling intensity, source-water chemistry, and stratification^91^

The evolution of marine N_2_O emissions in the twenty-first century in response to anthropogenic climate change remains uncertain^27–29^. While some models predict a decline in oceanic N_2_O emissions by 2100^27,28^, this decline seems to be heterogeneous across the global ocean, with N_2_O emissions in the eastern South Pacific predicted to enhance^27^. Uncertainties arise because although more fixed N loss from water column denitrification in expanded OMZs is expected, it is counteracted by less benthic denitrification due to the stratification-induced reduction in organic matter export^27,29^.

Given the ecological and socio-economic significance of EBUS, precise projections of fixed N loss, variability in DIN and in water columns are essential. Consequently, it is crucial to gain a comprehensive understanding of the impacts of hypoxia/suboxia and upwelling intensification on marine ecosystems and coastal communities. To achieve this, further research, long-term monitoring, enhanced data collection, and more sophisticated modeling efforts are indispensable.

### Supplementary Information

Supplementary Information.

## Data Availability

All data that support the findings of this study are included within the article (and any supplementary files). In addition, part of data of TSS are found in Farías, Laura; Quiñones Bergeret, Renato; Tapia, Fabián J (2023): A time series study of nitrous oxide distribution and ancillary variables off Conception, Chile (2016–2020). PANGAEA, 10.1594/PANGAEA.957416.
