# Supplementary material for: Understanding the impacts of coastal deoxygenation in nitrogen dynamics: an observational analysis
Source: Sci Rep. 2024 May 23;14:11826. doi: 10.1038/s41598-024-62186-w (PMC11116492; doi:10.1038/s41598-024-62186-w)
Supplement: Supplementary file 1 — Supplementary Information. [file 41598_2024_62186_MOESM1_ESM.pdf]

Supplementary materials

Understanding the impacts of coastal deoxygenation in nitrogen dynamics: An observational analysis

Farias, Laura[1,2,3] and de la Maza, Lucas[2,3].

Supplementary figures

Figure Supplementary 1. Continental shelf off Concepcion (central Chile) with bathymetry lines and location of time series station (TSS. 18), as well as the meteorological station of Carriel Sur. The pixel where ERA5-Winds data used to compare with data from continental station.

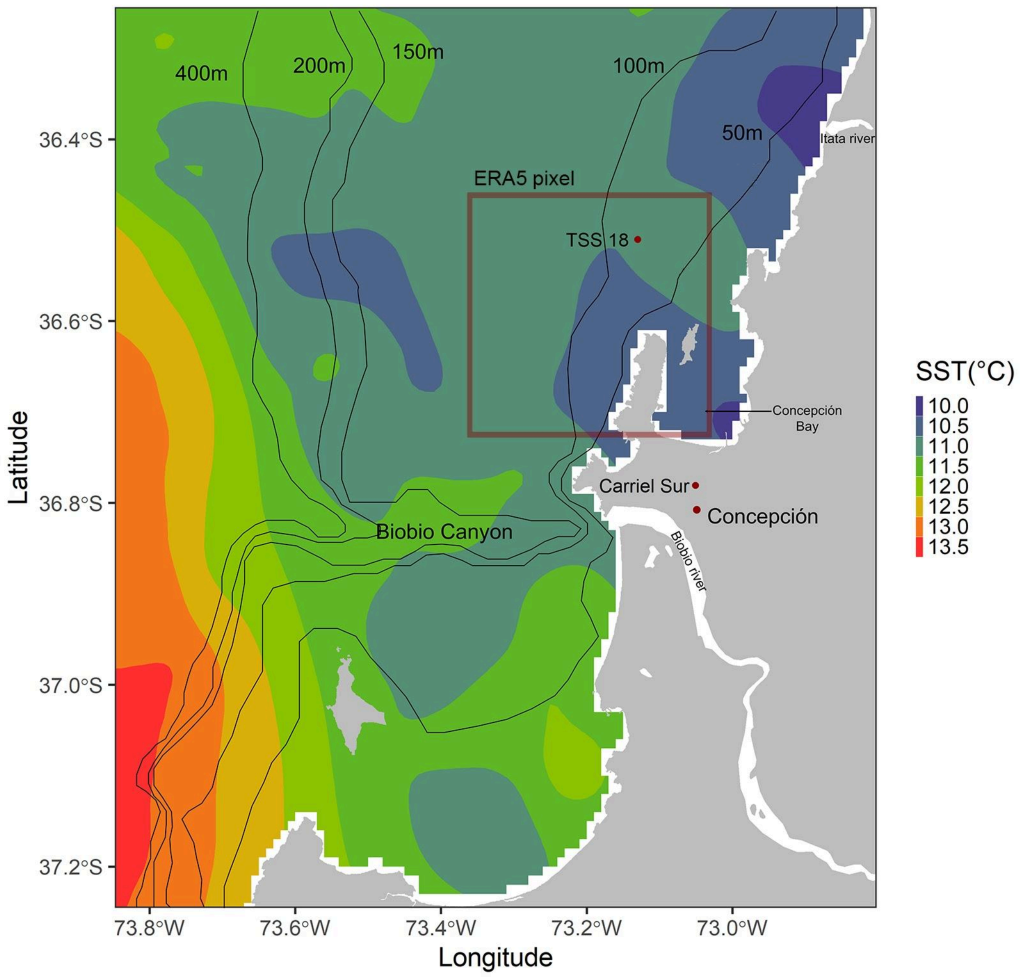

Figure Supplementary 2. Nitrate (a), nitrite (b) and Nitrous Oxide (c) concentrations plotted against dissolved oxygen. Dashed line represents DO<22 umol L^{-1}

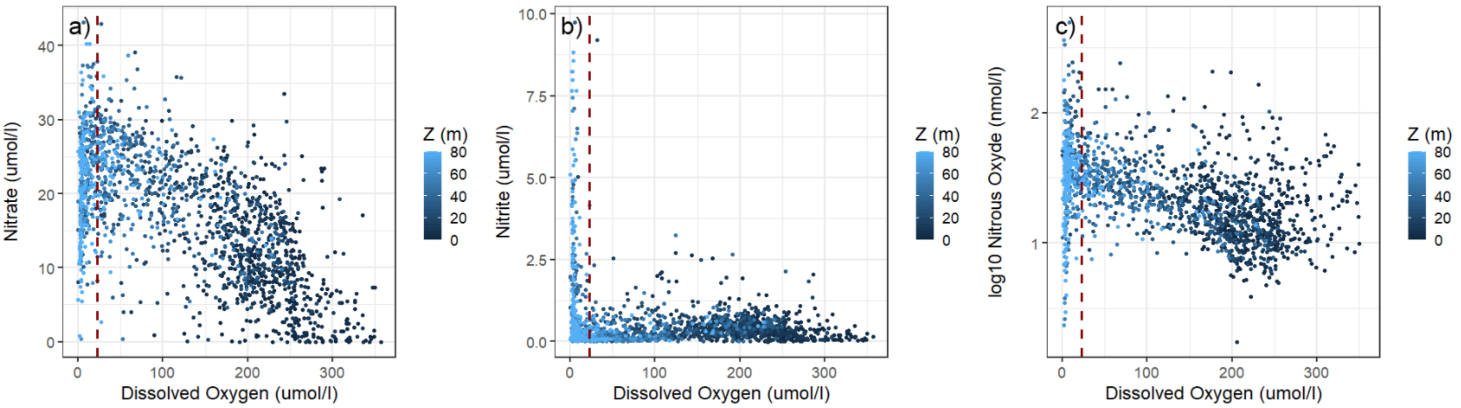

Figure Supplementary 3. Time series of El Niño (a) and La Niña (b) episodes from 1997 to 2023, categorized as central pacific or eastern pacific variants according to the methodology proposed by (Yang et al 2022) projected over the Oceanic Niño Index where El niño ≥ + 0.5 °C or ONI ≤ −0.5 °C La niña.

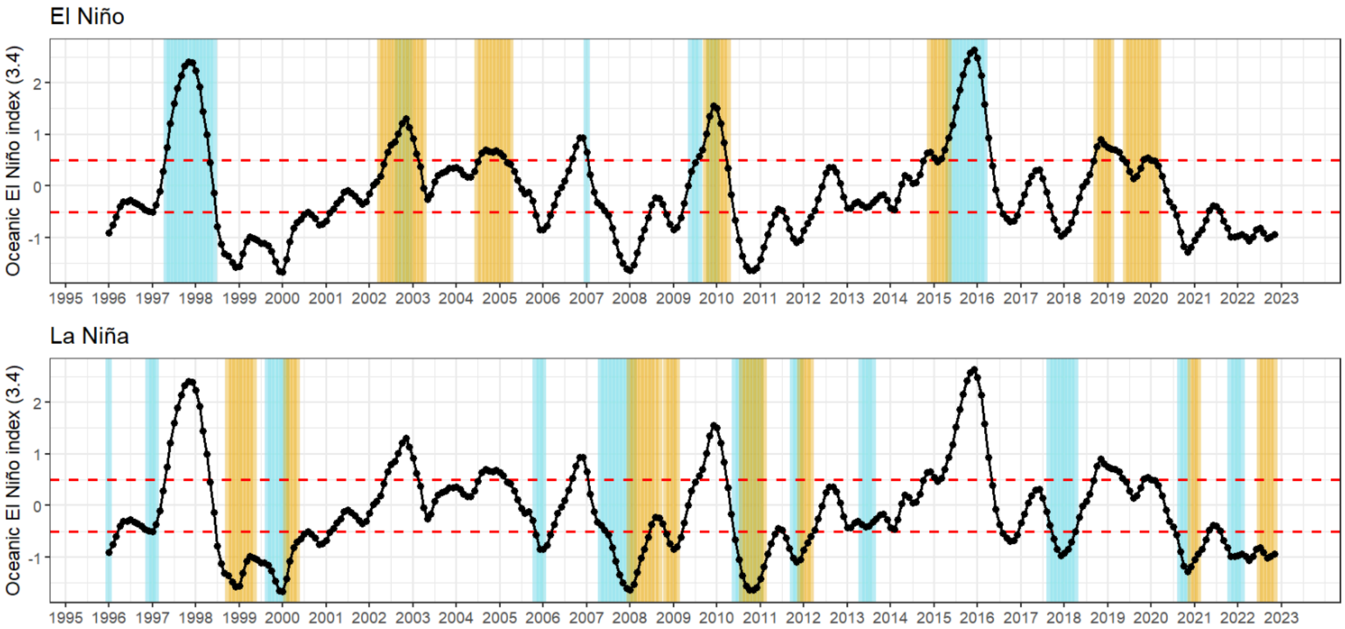

Figure Supplementary 4 The heatmap of correlation coefficient matrix among ONI and ICEN indices and physical (T°C, S, DO) and biogeochemical variables.

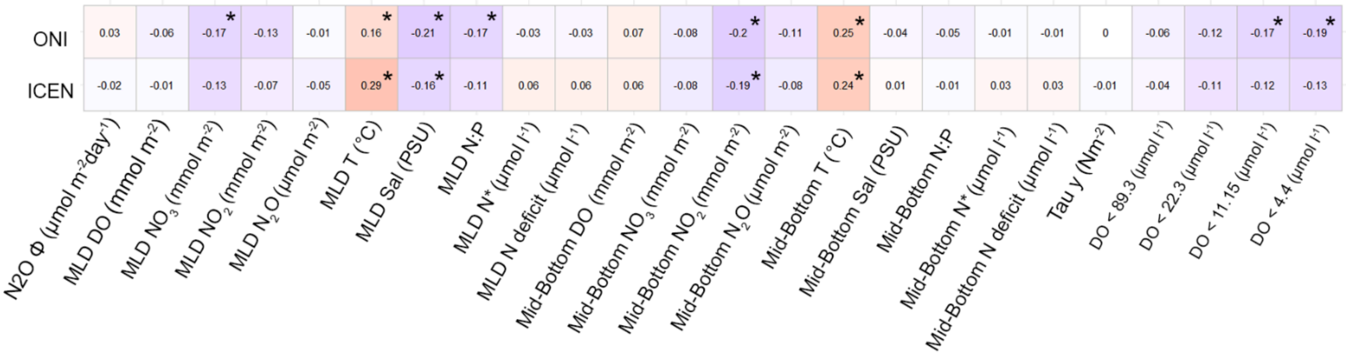

Figure Supplementary 5. First PC covariance structures for the (a) Upwelling, (b) Downwelling and (c) Aggregated cycles. Variables ordered from left to right in order of ponderation coefficient given by the eigenvectors (i.e. the higher the absolute value, the more important the variable is within the pattern).

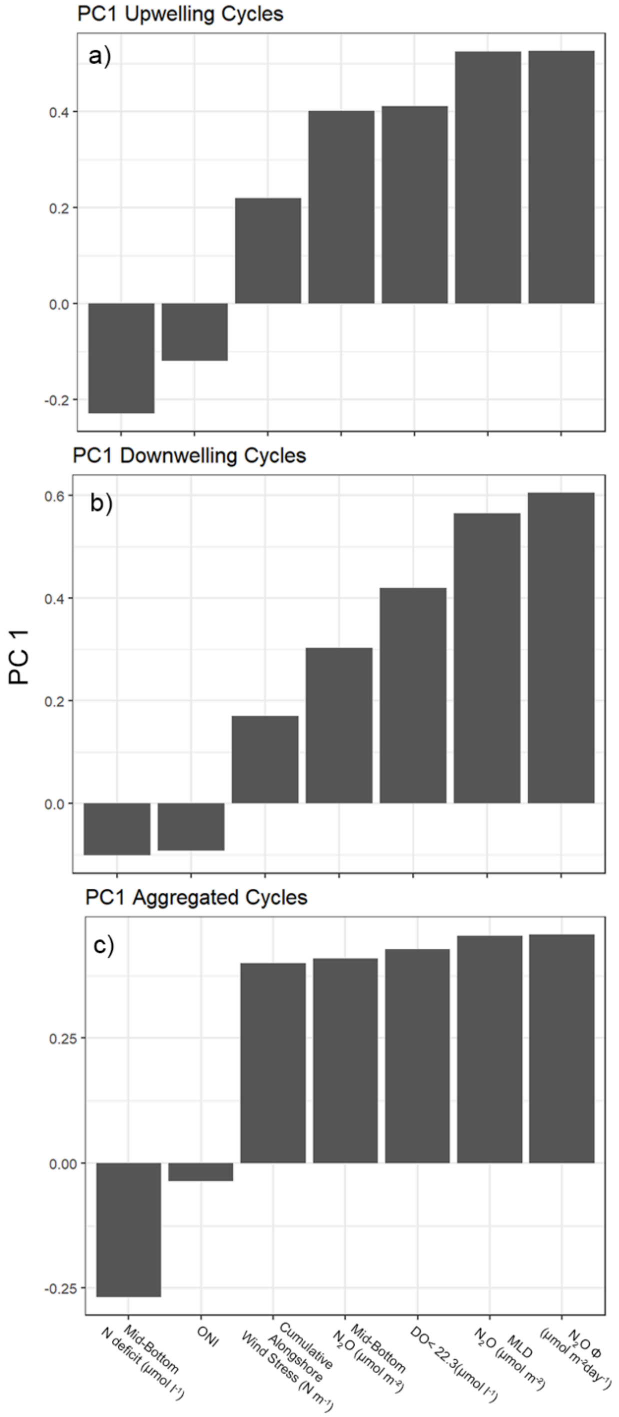

Figure Supplementary 6. Correlations of annually averaged variables with N_2O fluxes for Upwelling and Downwelling cycles and the aggregated cycles. Asterisks represent statistical significance.

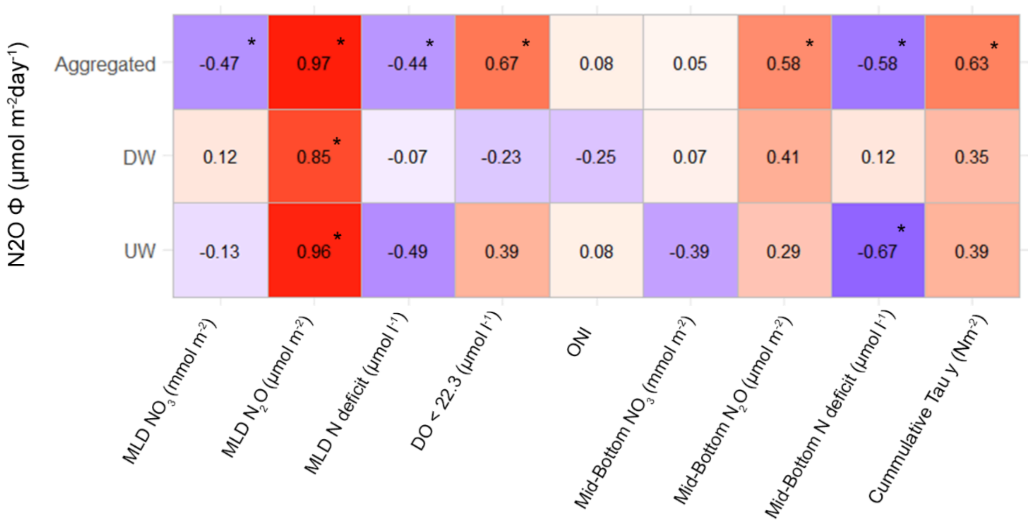

Supplementary Tables

Table S1 Monthly averages of inventories of nitrous oxide, nitrate and nitrite, N deficit, N_2O flux and cumulated wind stress estimated during the upwelling season (spring–summer) and non-upwelling season in both the mixed and subsurface layers, over 2000 and 2023 at TSS18.

	MLD (0-10m)		Mid-Bottom (30-80m)											
Cycle	NO_3 (mmol m^{-2}) Inventory	N_2O (µmol m^{-2}) Inventory	NO_3 (mmol m^{-2}) Inventory	N_2O (µmol m^{-2}) Inventory	N* (µmol l^{-1}) mean	N deficit (µmol l^{-1}) mean	N_2O flux (µmol $m^{-2}d^{-1}$)	N_2O Flux median	n	242 days Cumulative Wind Stress (N m^{-2})	O2<22.3 (µmol l^{-1}) mean	ONI	ICEN	|ONI| > 0.5
UP 2002-2003	64.46	144.62	827.03	736.84	-19.47	-22.37	6.82	3.36	10	0.49	0.29	0.68	0.08	*
DW 2003	168.24	93.67	906.88	710.65	-13.55	-16.45	-0.20	-0.22	5	-0.96	0.22	-0.01	-0.85	
UP 2003-2004	63.12	174.73	853.92	1203.33	-17.67	-20.57	6.02	2.87	9	0.72	0.24	0.29	-0.11	
DW 2004	140.94	146.26	841.48	1374.22	-13.09	-15.99	1.73	1.50	4	-0.64	0.09	0.39	-0.86	
UP 2004-2005	124.47	382.39	949.06	1933.68	-16.33	-19.23	19.31	10.93	8	1.03	0.55	0.60	-0.24	*
DW 2005	127.16	94.58	666.70	844.31	-9.37	-12.27	-0.24	-0.69	3	-1.84	0.13	0.03	-0.45	
UP 2005-2006	68.10	358.98	790.15	1252.22	-16.82	-19.72	16.29	8.71	9	1.35	0.37	-0.52	-0.71	*
DW 2005	97.75	88.13	678.53	631.78	-4.43	-7.33	-0.60	-1.00	3	-1.27	0.09	0.06	0.05	
UP 2006-2007	100.44	131.54	1082.02	1021.57	-7.58	-10.48	2.71	2.54	8	1.11	0.40	0.45	0.28	
DW 2007	193.95	111.12	923.32	962.72	-3.79	-6.69	0.62	0.86	4	0.24	0.05	-0.55	-1.26	*
UP 2007-2008	133.54	188.58	970.49	1729.01	-8.19	-11.09	5.86	3.05	7	1.77	0.28	-1.37	-0.66	*
DW 2008	156.65	84.64	829.57	1123.99	-10.62	-13.52	-0.08	-0.08	2	-0.75	0.11	-0.72	0.70	*
UP 2008-2009	101.86	288.85	923.07	1965.42	-14.52	-17.42	13.14	9.65	8	2.18	0.43	-0.56	-0.05	*
DW 2009	191.69	110.18	873.84	1145.47	-13.56	-16.46	0.43	0.19	4	0.66	0.23	0.33	0.69	
UP 2009-2010	120.51	380.11	939.30	1869.75	-17.23	-19.41	30.12	7.97	4	2.46	0.49	1.03	0.33	*
DW 2010	154.59	102.93	654.64	645.60	-20.48	-20.46	0.75	0.24	4	0.58	0.06	-0.81	-0.48	*
UP 2010-2011	89.98	246.02	672.05	1214.00	-22.02	-24.92	16.85	13.32	8	2.19	0.38	-1.34	-0.78	*
DW 2011	143.85	168.57	665.72	1070.17	-9.87	-12.77	4.54	4.78	4	0.74	0.06	-0.52	0.07	*
UP 2011-2012	139.93	256.66	1008.60	2407.02	-13.49	-16.39	11.37	4.92	8	2.06	0.42	-0.83	-0.16	*
DW 2012	118.73	97.79	698.39	723.20	-8.60	-11.50	0.06	0.10	3	0.16	0.04	0.20	0.53	
UP 2012-2013	124.25	141.65	790.93	1228.84	-15.70	-18.60	3.23	1.20	5	1.84	0.25	-0.18	-0.66	
DW 2013	159.18	139.21	601.04	860.92	-15.48	-18.38	1.70	1.42	4	0.45	0.13	-0.37	-1.44	
UP 2013-2014	88.57	258.14	839.79	1278.10	-13.23	-16.13	10.03	8.59	8	2.17	0.45	-0.25	-0.39	
DW 2014	115.02	72.88	636.26	675.42	-18.62	-21.52	-2.51	-1.44	4	0.29	0.06	0.12	1.09	
UP 2014-2015	45.22	612.42	665.89	1516.33	-23.34	-26.24	45.60	4.25	7	2.14	0.35	0.54	0.51	*
DW 2015	145.99	107.75	789.96	869.09	-11.25	-14.15	0.30	0.43	3	0.56	0.05	1.32	2.01	*
UP 2015-2016	50.98	138.95	733.23	1251.76	-10.32	-13.22	3.47	2.44	8	2.22	0.21	2.12	1.77	*
DW 2016	111.18	210.70	781.28	711.51	-13.13	-16.03	2.50	0.84	3	0.34	0.00	-0.32	0.45	
UP 2016-2017	92.04	207.77	828.29	1599.91	-6.96	-9.86	7.62	8.50	4	2.48	0.46	-0.35	0.75	
DW 2017	103.90	121.29	555.89	877.71	-8.32	-11.22	2.64	0.62	4	0.00	0.01	0.16	-0.07	
UP 2017-2018	81.82	223.19	659.04	1603.90	-17.69	-20.59	10.10	9.83	4	2.19	0.43	-0.66	-0.99	*
DW 2018	91.38	134.92	535.72	1128.26	-9.36	-12.26	1.89	2.06	4	0.37	0.22	0.02	-0.50	
UP 2018-2019	80.33	228.25	744.34	1256.55	-14.64	-17.54	11.19	10.03	6	2.45	0.35	0.76	0.55	*
DW 2019	101.81	144.74	676.44	1213.43	-14.39	-17.29	2.85	1.60	4	0.40	0.16	0.35	-0.29	
UP 2019-2020	87.99	291.69	617.05	1872.46	-26.25	-28.41	17.39	8.24	7	2.69	0.44	0.38	-0.27	
DW 2020	125.11	115.10	661.13	704.04	-15.83	-18.73	1.02	1.31	4	0.64	0.00	-0.34	-0.64	
UP 2020-2021	69.81	261.32	836.83	1539.52	-17.41	-20.31	16.41	9.73	8	2.78	0.41	-1.05	-0.84	*
DW 2021	101.45	142.91	744.20	1211.05	-15.47	-18.37	3.22	2.18	3	-0.38	0.38	-0.42	-0.38	
UP 2021-2022	149.65	253.61	782.28	1955.97	-15.30	-18.20	14.80	15.40	8	1.55	0.44	-0.93	-1.08	*
DW 2022	136.76	118.61	756.94	958.55	-9.19	-12.09	0.52	0.29	4	-0.46	0.15	-0.89	-1.17	*
UP 2022-2023	141.49	395.35	792.54	2850.93	-11.29	-14.19	31.41	27.92	5	1.55	0.48	-0.69	-0.34	*

Supplementary materials
Extended Materials and methods

N_2O air-sea fluxes were estimated monthly using discrete gas measurements on the mixed layer; considering the difference between the mean N2O concentration in the mixed layer (C_w) and N_2O concentration in the mixed layer depth (MLD) expected to be in equilibrium with the atmosphere (C_{eq}) according to Weiss and Price (1980). Transfer gas velocity (kw) as a function of wind speed parametrization was based on Wanninkhof (2014) or W2014 and compared with that of Wanninkhof (1992) or W92. Atmospheric N_2O concentration at each sampling time was obtained from the NOAA Earth System Research Laboratories program (NOAA/ESRLprogram: http://www.esrl.noaa.gov/gmd/hats/combined/N2O.html), assuming a well-mixed atmosphere with no major latitudinal variations (Bange et al 2010). The MLD was calculated using a potential density-based criterion as the MLD varied from 12 to 17 m depth (with mean±SD=10.7±3.35) (Testa et al 2018); thus, an average of N_2O levels at 1, 5 and 10 m depth was considered as surface ocean N_2O concentration. Wind speed and direction, based on hourly register, were obtained from a permanent meteorological station located at Carriel Sur (http://www.meteochile.gob.cl/) and to calculate fluxes the wind speed mean of the 7 days window preceding each estimate was used.

These data were compared with those three-hour vector wind data obtained from the ERA5, which is the fifth generation ECMWF (European Center for Medium-Range Weather Forecasts) atmospheric reanalysis of the global climate, and available at the Copernicus Climate Change Service Information (2019) https://cds.climate.copernicus.eu/cdsapp#!/home). This product has a spatial resolution of ~0.25° and here the pixel used for the analysis was the closest one to the TSS 18 centered at 36.6°S and 73.2°W. The correlation between winds measured by Carriel Sur and ERA5 is 0.75 ($n=7545$; $p<0.05$), the variability in the winds of Carriel Sur is 30% less than that of ERA5 at the selected pixel while the correlation between N2O flux estimates done with the ERA5 and the ones obtained using the meteorological station at Carrier Sur was 0.98 ($n = 221$; $p<0.05$) and the meteorological station estimates are dampened by 50% in relation to ERA5.

Climatologies were calculated by the Fast Fourier transform method fitting the seasonal frequency harmonics for each variable obtained in the TSS. This is the optimal method over plain averaging of the data (Narapusetty et al 2009). Then the climatology was subtracted from the time series to obtain the anomaly. N_2O concentration presents high variability, in some cases exceeding by an order of magnitude its usual variability, these events are denominated hotspots (Farias et al 2015) and were determined as standardized N_2O levels exceeding 2 standard deviation units in relation to the time series absolute mean. Trends and basic statistics were calculated with and without these extreme events to assess their impact on the observed long term patterns.

To quantify the proportion of hypoxic waters present at the sampling location 4 thresholds were defined, to then identify which oxygen isoline was associated with higher N2O concentrations,

these thresholds were 89.3 umol/l; 22.3 umol/l; 11.15 umol/l and 4.4 umol/l. It is noteworthy that these DO levels are coarse and may be lower if they were measured with a more sensitive sensor (Revsbech et al 2009).

We estimated the proportion of hypoxic waters as:

$$Hypoxic\,proportion = H = \frac{hh}{ht}$$

where hh is the depth (length) of hypoxic waters defined by the shallowest register of oxygen below one of the thresholds and ht is the total depth of the measured water column.

All the statistical analyses were conducted using R version 4.2.1. To determine the relative importance of different variables required to explain N_2O air-sea flux and N deficit in the water column, multiple pairwise correlation analyses using the Pearson correlation test were performed. The threshold value for statistical significance was set at $p < 0.05$. This allowed us to determine a first-order linear relationship between different potential drivers. We analyzed N_2O fluxes and N deficit as core estimates that depend on temperature, salinity, winds (wind stress), inorganic nutrients concentration and its ratio, and inventories per layers: (1) surface (0, 5 and 10 m depth) and (2) mid- bottom (from 30 to 80 m depth) of N_2O, NO_3^-, NO_2^- and DO were performed by numerical integration of data at one meter increments (linear interpolation) based on at least 3-6 sampled depth per layer. For fair comparison across variables, we removed outliers from the dataset based on the expected ranges of concentration according to water mass structure in the area.

Regarding interannual variability, ENSO and oceanic episodes of El Niño (EN) and La Niña (LN) were detected using two indexes, the Oceanic Niño Index (ONI) which is based on the temperature anomaly along the Central Equatorial Pacific Ocean (3.4 region 5°N–5°S, 120°–170°W) and the coastal El Niño index (ICEN) which is based on the temperature anomaly off the coast of Ecuador and Perú (1 + 2 region 90°−80°W, 10°S–0°). In both cases, negative and positive values indicate an LN and EN episodes, respectively (Takahashi et al 2014). The data was obtained from https://origin.cpc.ncep.noaa.gov/products/analysis_monitoring/ensostuff/ONI_v5.php for ONI and from http://met.igp.gob.pe/datos/icen.txt for ICEN.

For Figure S3, the central pacific and eastern pacific variants of EN and LN were identified according to the methodology in Yang et al 2022.

In addition to assess variability among years, the dataset from 2002 to 2023 was divided into 21 cycles, from September to March (upwelling favorable season) and from April to August (non-upwelling season). Cumulative alongshore (south–north) wind stress for each cycle was obtained from the cumulative sum of wind stress from the start to finish of each cycle (Farias et al 2015). Wind stress was calculated according to Nelson et al 1977 as:

$$\tau_y = C_d \cdot \rho_{air} \cdot |V| \cdot v$$

Where: C_d is the drag coefficient of wind at 10 meters above sea level and corresponds to the value of 0.0013, ρ_{air} is the air density (1.22 kg m^{-3}), $|V|$ is the wind speed (m s^{-1}) and *v* is the meridional component of the wind speed (m s^{-1}).

To determine the primary drivers of interannual variability in N_2O sea-air flux, a principal components analysis (PCA) methodology was employed. This involved generating a correlation matrix based on annually averaged time series data encompassing mixed layer N_2O, mid-bottom layer N_2O, N_2O fluxes, N deficit, proportion of hypoxic waters (DO <22.3 umol/l), alongshore wind stress, and ONI index values. The principal components were derived and subsequently, the variance attributable to each original variable within every principal component was computed. This approach differentiates from the conventional PCA, wherein the aggregate variance within each component typically serves as the outcome of interest. In this case the variable of interest is N2O flux. Thus the aim of the analysis is to identify which ones of the original variables dominate (are the most relevant) the Principal Component that contains most of the variance of N2O flux. This analysis was performed for the time series divided into upwelling and downwelling seasons as well as the aggregated seasons (September to August).

References not cited in-text on the main text but relevant in these extended methods:

Takahashi, K., Mosquera-Vásquez, K. A. and Reupo, J. El Índice Costero El Niño (ICEN): historia y actualización. (Boletín Técnico Generación de modelos climáticos para el pronóstico de la ocurrencia del Fenómeno El Niño. ed. K. Takahashi, Lima: Instituto Geofísico del Perú), 8–9, http://hdl.handle.net/20.500.12816/4639 (2014)

Yang, X., Song, Y., Wei, M., Xue, Y. and Song, Z. Different Influencing Mechanisms of Two ENSO Types on the Interannual Variation in Diurnal SST over the Niño-3 and Niño-4 Regions. J. Climate, 35, 125–139; 10.1175/JCLI-D-20-0815.1. (2022)

Narapusetty, B., Del Sole, T. and Tippett, M. K. Optimal Estimation of the Climatological Mean. J. Climate, 22, 4845–4859; 10.1175/2009JCLI2944.1. (2009)

Revsbech, N. P., Larsen, L. A., Gundersen, J., Dalsgaard, T., Ulloa, O. and Thamdrup, B. Determination of ultra-low oxygen concentrations in oxygen minimum zones by the STOX sensor. Limnol. Oceanogr. Methods, 7, 371-381; 10.4319/lom.2009.7.371 (2009)
